# Notes on *Allium* section *Rhizirideum* (Amaryllidaceae) in South Korea and northeastern China: with a new species from Ulleungdo Island

**DOI:** 10.3897/phytokeys.176.63378

**Published:** 2021-04-16

**Authors:** Ju Eun Jang, Jong-Soo Park, Ji-Young Jung, Dong-Kap Kim, Sungyu Yang, Hyeok Jae Choi

**Affiliations:** 1 Department of Biology and Chemistry, Changwon National University, Changwon 51140, South Korea; 2 Division of Forest Biodiversity and Herbarium, Korea National Arboretum, Pocheon 11186, South Korea; 3 Division of Plant Resources, Korea National Arboretum, Pocheon 11186, South Korea; 4 Herbal Medicine Resources Research Center, Korea Institute of Oriental Medicine, Naju 58245, South Korea

**Keywords:** Chromosome number, DNA barcode, distribution, morphology, new species, synonym, taxonomy

## Abstract

Allium section Rhizirideum is reviewed for South Korea and neighboring northeastern China based on critical observation of wild populations and herbarium materials. Species delimitations are re-evaluated on the basis of morphological and somatic chromosome numbers, resulting in the recognition of five species. *Allium dumebuchum* from Ulleungdo Island, South Korea, is described as a new species. This species is most similar to *A. senescens* due to its habits, but is clearly distinguished particularly by its rhomboid scapes in cross-secion, light purple perianth color, entire and narrowly triangular inner filaments, and flowering season from late September. One previously recognized species is placed into synonymy: *A. pseudosenescens* (under *A. senescens*). Photographs and a key to species of Allium section Rhizirideum in South Korea and northeastern China are provided in addition to information on nomenclatural types, synonymies, chromosome numbers, distribution, and specimens examined.

## Introduction

With over 900 species (Seregin et al. 2015), *Allium* L. is one of the largest genera in the Amaryllidaceae (Friesen et al. 2006; Fritsch et al. 2010; Li et al. 2010). It is characterized by bulbs enclosed in membranous to fibrous tunics, free or almost free tepals, and often a subgynobasic style (Friesen et al. 2006). Most taxa produce remarkable amounts of cysteine sulphoxides causing the well-known characteristic odor and taste (Friesen et al. 2006). *Allium* is distributed naturally in the northern hemisphere and in South Africa, mostly in regions with dry seasons (De Sarker et al. 1997; Friesen et al. 2006; Nguyen et al. 2008; Neshati and Fritsch 2009). The classification of *Allium* by Friesen et al. (2006) based on molecular phylogenetic analyses includes 15 subgenera and 56 sections. About 23 taxa, excluding cultivated species, are known from the Korean peninsula and neighboring northeastern China (Choi and Oh 2011; Shukherdorj et al. 2018; Choi et al. 2019).

Allium section Rhizirideum G.Don ex W.D.J.Koch is the typical section of subgenus Rhizirideum (G.Don ex W.D.J.Koch) Wendelbo and characterized by having bulbs enclosed in membranous tunics and attached to horizontal rhizomes, a leaf shape ranging from hemicylidrical to plain, and a flower color from white to purple (Sinitsyna et al. 2016). Section Rhizirideum consists of 24 species and is part of the third of three main evolutionary lines of *Allium* (Fritsch 2001; Fritsch and Friesen 2002; Friesen et al. 2006; Li et al. 2010; Choi et al. 2012b). The distribution area of section Rhizirideum reaches from Europe to East Asia (Sinitsyna et al. 2016). There is a distinct narrowing of the distribution area east of the Ural Mountains approximately along 70° eastern longitude, and most species of section Rhizirideum are distributed in temperate Asia (Sinitsyna et al. 2016). The center of species diversity is situated in the mountain steppes of South Siberia and Mongolia (Sinitsyna et al. 2016). The species of section Rhizirideum share a basic chromosome number of *x* = 8, and four ploidy levels were found: di-, tetra- penta-, and hexaploids (Sinitsyna et al. 2016).

The taxonomy of the section is complicated because of morphological diversity and hybridization involving polyploidy (Friesen 1988, 1992; Kamelin 2004). Additionally, the nomenclature is confusing, which may be explained by similar morphology of some species and disappearance of many morphological characters in the voucher specimens in herbaria (Sinitsyna et al. 2016; Sinitsyna and Friesen 2018). Recently, Sinitsyna et al. (2016) and Sinitsyna and Friesen (2018) investigated the phylogenetic relationships in the section Rhizirideum based on molecular markers, and organized nomenclature, distribution maps and identification key for all known species of the section. Although there is general agreement regarding the Allium species of section Rhizirideum in South Korea and its neighboring northeastern China (Choi and Oh 2011) studies on materials from these regions are still limited.

Here, we have combined morphological, cytological, and molecular characters to address the taxonomy of Allium section Rhizirideum, and organized nomenclature, distribution maps and identification key for species in South Korea and north-eastern China. The goals of this study are: 1) to review and expand the current knowledge on general morphology (in addition to Choi and Oh 2011; Sinitsyna et al. 2016), somatic chromosome numbers (in addition to Choi and Oh 2011), DNA barcoding, and distribution (in addition to Choi and Oh 2011; Sinitsyna et al. 2016) especially with a focus on the materials from South Korea and north-eastern China, and 2) to describe a new species of section Rhizirideum from Ulleungdo Island, South Korea, *A. dumebuchum* H.J.Choi. This study together with that of Sinitsyna et al. (2016) and Sinitsyna and Friesen (2018) will provide a sound foundation for a global monograph and the systematic understanding of Allium section Rhizirideum.

## Materials and methods

### Morphological characters

This revision is based on the use of living and herbarium material, including photographs of type specimens, from the following herbaria: B, CBU, KB, KH, KWNU, LE, LINN, PE (abbreviations are according to Thiers 2020+), and the herbarium of Changwon National University (CWNU). Field surveys were carried out mainly in South Korea and north-eastern China from July 2014 to October 2020. We also observed populations from Far Eastern Russia and Mongolia especially for *Allium spirale* Willd. and *A. senescens* L. Materials preserved in 70% ethanol were used especially for observation and measurement of floral parts, cross-sections of leaf and scape. Segments from the middle third of the leaf blade and scape were stained with 2% aceto carmin for observation of the cross-section. Measurements were based on at least 30 samples for quantitative characters.

### Principal component analysis

To analyze floral morphology known as a key character to distinguish *Allium* species (Choi and Oh 2010; Choi and Oh 2011), principal components analysis (PCA) was performed based on 14 characters: flower number per inflorescence, inflorescence length, inflorescence width, pedicel length, inner tepal length, inner tepal width, outer tepal length, outer tepal width, inner filament length, inner filament width, outer filament length, anther length, anther width, and pistil length. The principal components analysis used the {ggfortify} and {ggplot2} packages of the R-project (Tang et al. 2016; Wickham 2016; R Core Team 2020). The specimens used for principal components analysis were indicated with an asterisk (*) in specimens examined for each species.

### Somatic chromosome numbers

Root tips were pre-treated in distilled water on ice for 24 h in total darkness at 4 °C and then fixed in Carnoy’s fluid (3 parts absolute ethanol: 1 part glacial acetic acid, v/v) overnight at 4 °C. The root tips were macerated in 1M hydrochloric acid at 60 °C for 3–5 min. After washing 3–5 times to eliminate residual hydrochloric acid and staining with feulgen for 5 min, the material was squashed for observation in 2% aceto carmin. Observations and photographing of chromosome micrographs were made using an Olympus BX43 (Tokyo, Japan).

### DNA barcoding

In this study, we investigated the application of concatenated cpDNA regions of *ndhJ*-*trnF*, *trnH*-*psbA*, *psbD*-*trnT*, and *psbJ*-*petA* in barcoding analyses of Allium section Rhizirideum and related taxa (Table 1). In order to analyze the relationship among the species using these four cpDNA regions, we extracted each cpDNA region from complete chloroplast genome sequences stored in NCBI GenBank (https://www.ncbi.nlm.nih.gov/; Fig. 6). The species in subgen. Butomissa were selected as outgroup referred from wide phylogenetic study of *Allium* (Li et al. 2010). Detailed information on sample collection, voucher specimens and Genbank accession numbers of each sample is provided in Table 2.

**Table 1.** List of the markers used for the DNA barcoding and phylogenetic analysis.

Fragment	Marker	Sequence 5’ → 3’	Reference
*ndhJ-trnF*	*ndhJ*	ATGCCYGAAAGTTGGATAGG	Shaw et al. (2007)
	TabE	GGTTCAAGTCCCTCTATCCC	Taberlet et al. (1991)
*trnH-psbA*	*trnH^GUG^*	CGCGCATGGTGGATTCACAATCC	Tate and Simpson (2003)
	*psbA*	GTTATGCATGAACGTAATGCTC	Sang et al. (1997)
*psbD-trnT*	*psbD*	CTCCGTARCCAGTCATCCATA	Shaw et al. (2007)
	*trnT^GGU^*-R	CCCTTTTAACTCAGTGGTAG	
*psbJ-petA*	*psbJ*	ATAGGTACTGTARCYGGTATT	Shaw et al. (2007)
	*petA*	AACARTTYGARAAGGTTCAATT	

Total genomic DNA was extracted from silica gel-dried leaf materials using the DNeasy Plant Mini Kit (Qiagen, Seoul, South Korea). We conducted PCR with a ProFlex 96-Well PCR System (Applied Biosystems, Foster City, CA, USA). Each reaction mixture contained AccuPower PCR PreMix (Bioneer, Daejeon, South Korea), ca. 10 ng (1μL) of genomic DNA, and 100 pM of primers in a total volume of 20 µL. Conditions included an initial denaturation at 94 °C for 5 min, followed by 30 amplification cycles comprising 94 °C for 1 min, 54 °C for 1 min, and 72 °C for 1 min, with a final extension at 72 °C for 7 min. After the PCR products were visualized on 2% agarose gels, they were treated with a MG PCR Purification kit (MGmed), and sequenced with the ABI 3730xl Analyzer, using the ABI BigDye Terminator v3.1 Cycle Sequencing Kits (Applied Biosystems, Foster City, CA, USA). The obtained sequences were manually determined and aligned by using MAFFT with Geneious Prime 2019.2.3 (Biomatters Ltd., Auckland, NZ). The DNA sequences generated in this study have been deposited in GenBank (Table 2).

**Table 2.** List of *Allium* species sequenced in this study.

Taxon	Locality	Voucher information	GenBank number			
			*psbJ*-*petA*	*ndhJ*-*tabE*	*psbA*-*trnH*	*psbD*-*trnT*
*A. angulosum*	Kazakhstan: Burlinsky, Zharsuat	*H.J.Choi 200923*	MW478175	MW478211	MW478247	MW478283
*A. austrosibiricum*	Mongolia: khovd, Munkhkhairkhan, Khuren khesuu	*H.J.Choi 160730-001*	MW478174	MW478210	MW478246	MW478282
	Mongolia: khovd, Munkhkhairkhan	*H.J.Choi 160730-002*	MW478173	MW478209	MW478245	MW478281
*A. dumebuchum*	South Korea: Gyeongbuk, Ulleungdo, Nari	*H.J.Choi 190917-01*	MW478172	MW478208	MW478244	MW478280
	South Korea: Gyeongbuk, Ulleungdo, Nari	*H.J.Choi 190917-02*	MW478171	MW478207	MW478243	MW478279
*A. minus*	South Korea: Gyeonggi, Yangju, Jangheung	*H.J.Choi 151006-01*	MW478170	MW478206	MW478242	MW478278
	South Korea: Gyeonggi, Yangju, Jangheung	*H.J.Choi 151006-02*	MW478169	MW478205	MW478241	MW478277
*A. prostratum*	Mongolia: Ulaanbaatar, Uvor Gunt davaa	*H.J.Choi 140708*	MW478168	MW478204	MW478240	MW478276
	Mongolia: Govi-Altai	*H.J.Choi 160811*	MW478167	MW478203	MW478239	MW478275
*A. senescens*	Mongolia: Ulaanbaatar, Sanzai	*2014-MON-010*	MW478166	MW478202	MW478238	MW478274
	Mongolia: Tuv, Mungunmorit	*H.J.Choi 160706*	MW478165	MW478201	MW478237	MW478273
*A. spirale*	Russia: Primorskiy kray, Terneysky	*H.J.Choi et al. 140826-01*	MW478157	MW478193	MW478229	MW478265
	Russia: Primorskiy kray, Terneysky	*H.J.Choi et al. 140826-02*	MW478156	MW478192	MW478228	MW478264
	Russia: Primorskiy kray, Khasansky, Schultz	*H.J.Choi et al. 150819-01*	MW478153	MW478189	MW478225	MW478261
	Russia: Primorskiy kray, Khasansky, Schultz	*H.J.Choi et al. 150819-02*	MW478152	MW478188	MW478224	MW478260
	Russia: Primorskiy kray, Sukhanovka	*H.J.Choi et al. 150817-01*	MW478155	MW478191	MW478227	MW478263
	Russia: Primorskiy kray, Sukhanovka	*H.J.Choi et al. 150817-02*	MW478154	MW478190	MW478226	MW478262
	Russia: Primorskiy kray, Khasansky	*2015RUSV017-01*	MW478164	MW478200	MW478236	MW478272
	Russia: Primorskiy kray, Khasansky	*2015RUSV017-02*	MW478163	MW478199	MW478235	MW478271
	South Korea: Gangwon, Goseong	*H.J.Choi 191010-01*	MW478159	MW478195	MW478231	MW478267
	South Korea: Gangwon, Goseong	*H.J.Choi 191010-02*	MW478158	MW478194	MW478230	MW478266
	South Korea: Gangwon, Gangneung	*H.J.Choi 190919-001-01*	MW478162	MW478198	MW478234	MW478270
	South Korea: Gangwon, Gangneung	*H.J.Choi 190919-001-02*	MW478161	MW478197	MW478233	MW478269
	South Korea: Gangwon, Goseong	*NAPI-10-139-01*	MW478151	MW478187	MW478223	MW478259
	South Korea: Gangwon, Goseong	*NAPI-10-139-02*	MW478150	MW478186	MW478222	MW478258
	South Korea: Gangwon, Goseong	*NAPI-10-139-03*	MW478149	MW478185	MW478221	MW478257
	South Korea: Gangwon, Gangneung	*H.J.Choi 190919-002*	MW478160	MW478196	MW478232	MW478268
*A. spurium*	South Korea: Gyeongbuk, Bonghwa, Cheongnyangsan	*H.J.Choi 200831-01*	MW478144	MW478180	MW478216	MW478252
	South Korea: Gyeongbuk, Bonghwa, Cheongnyangsan	*H.J.Choi 200831-02*	MW478143	MW478179	MW478215	MW478251
	China: Jilin, Erdaobaihe	*H.J.Choi 190908-001-01*	MW478146	MW478182	MW478218	MW478254
	China: Jilin, Erdaobaihe	*H.J.Choi 190908-001-02*	MW478145	MW478181	MW478217	MW478253
	China: Jilin, Linjiang	*H.J.Choi 190429-01*	MW478148	MW478184	MW478220	MW478256
	China: Jilin, Linjiang	*H.J.Choi 190429-02*	MW478147	MW478183	MW478219	MW478255
*A. thunbergii*	South Korea: Gangwon, Goseong	*H.J.Choi 190901*	MW478142	MW478178	MW478214	MW478250
*A. tuberosum*	China: Jilin, Erdaobaihe	*H.J.Choi 190908-002-01*	MW478141	MW478177	MW478213	MW478249
	China: Jilin, Erdaobaihe	*H.J.Choi 190908-002-02*	MW478140	MW478176	MW478212	MW478248

The phylogenetic analyses were conducted using Maximum Likelihood (ML) by using W-IQ-TREE (Trifinopoulos et al. 2016), based on user-friendly web servers for IQ-TREE (Nguyen et al. 2015). The concatenated sequence dataset was tested to find the best-fit model by using W-IQ-TREE with the Akaike criterion and new model selection procedures. TIM+R3+F were confirmed as best-fit models for the sequences. Maximum likelihood analysis was performed with default settings in W-IQ-TREE (Fig. 6).

## Results

### Morphological characters

**Figure 1.**
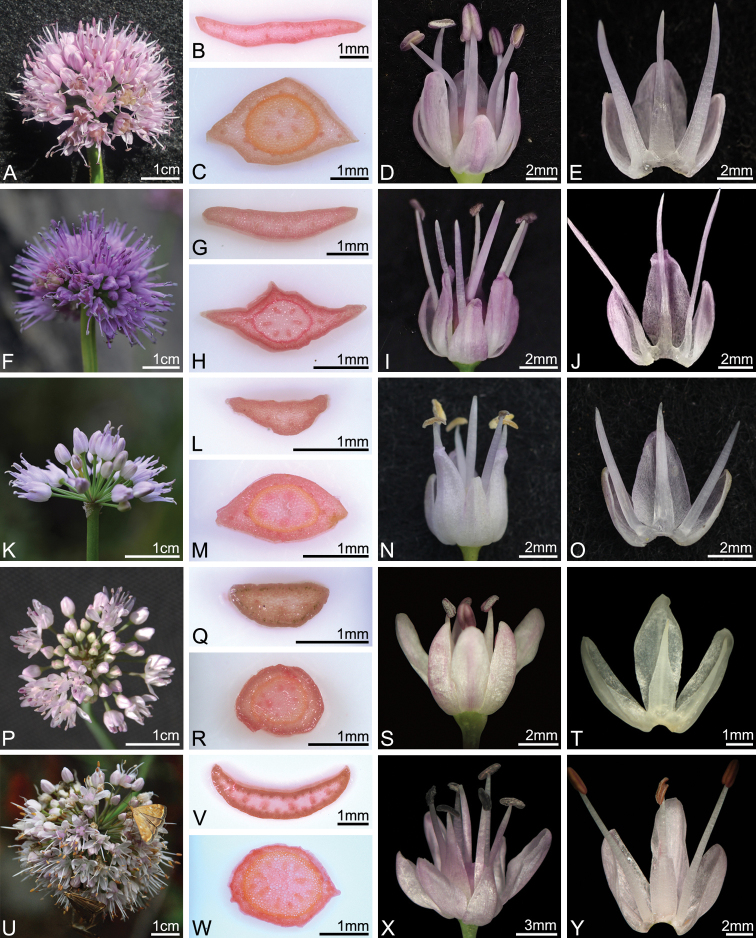
Comparative photographs of the inflorescence, cross-section of leaf and scape, flower, and tepal and filament arrangement of Allium section Rhizirdeum in South Korea and northeastern China **A–E***A. dumebuchum* (*H.J.Choi 201008-001*) **F–J***A. spirale* (*H.J.Choi 191010-01*) **K–O***A. spurium* (*H.J.Choi 200831-01*) **P–T***A. minus* (*H.J.Choi 080063*) **U–Y***A. senescens* (*H.J.Choi 080119*).

**Figure 2.**
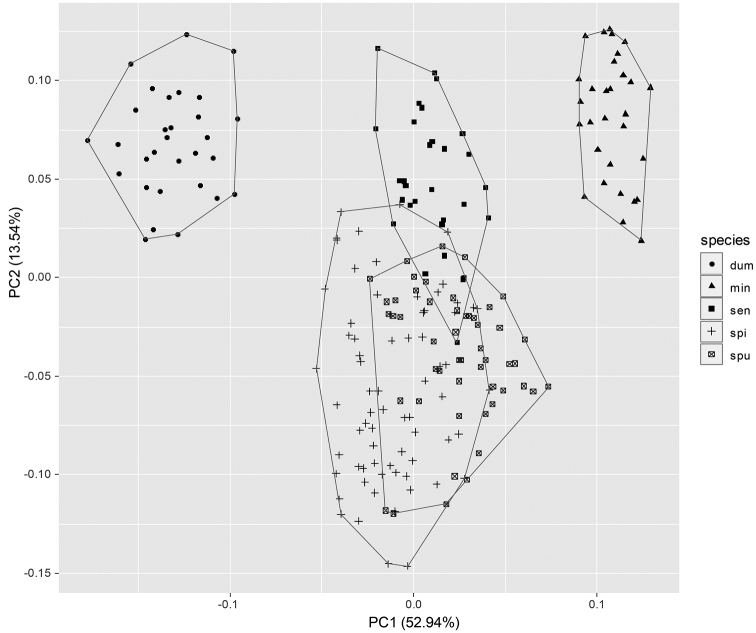
Principal components analysis plot of five Allium species of section Rhizirideum in South Korea and northeastern China. dum = *A. dumebuchum*; min = *A. minus*; sen = *A. senescens*; spi = *A. spirale*; spu = *A. spurium*.

Our data indicate that several morphological characters are of taxonomic utility in Allium section Rhizirideum. Among these, the shape and size of leaf, scape and various floral parts are useful diagnostic traits at the specific level (Table 3; Fig. 1; Choi and Oh 2010; Choi and Oh 2011). According to the PCA results, first combined five principal components accounted for 83.65% of the total variation among traits in the studied taxa. The PC1 accounted for 52.94% of variance, while PC2 accounted for 13.54% of total variability. The first two principal components were strongly associated with the inflorescence length, outer tepal length and inner filament width. The anther length and inner tepal width were mostly contributed to PC1, while the pedicel length and flower number were contributed only to PC2. PC1 versus PC2 in scatter plot showed that *A. dumebuchum* and *A. minus* were distinctly separated from *A. senescens*, *A. spirale*, and *A. spurium* (Fig. 2).

**Table 3.** Comparison of major characters of Allium section Rhizirideum in South Korea and northeastern China.

Character		*A. dumebuchum*	*A. spirale*	*A. spurium*	*A. minus*	*A. senescens*
Rhizome		oblique to horizontal	horizontal	horizontal	oblique	horizontal
Leaf sheath		exposed	buried	buried	exposed	exposed
Leaf blade	texture	fleshy, glaucous	leathery, lustrous	leathery, lustrous	fleshy, glaucous	fleshy, glaucous
	length (cm)	19.5–38.0	20.0–45.0	15–30.0	11.4–24.5	23.0–45.0
	width (mm)	3.8–13.0	4.0–10.0	1.5–4.0	2.8–4.5	5.0–15.0
Scape	cross-section	rhomboid	flattened-winged	rhomboid to subterete	subterete	subterete
	length (cm)	23.4–49.0	33.0–65.0	10.0–40.0	11.7–20.5	25.8–70.0
	diameter (mm)	2.5–5.6	4.0–5.1	1.5–2.5	1.5–1.6	3.0–5.5
Pedicel	length (mm)	9.8–11.2	6.0–12.4	7.6–11.1	8.7–11.1	8.0–13.0
Perianth	shape	semi-radially spreading	campanulate	campanulate	radially spreading	radially spreading
	color	light purple	reddish purple	strong purple or pale purple	pale purple	pale purple
Inner tepal	shape	elliptical to ovately-elliptical	ovately-elliptical	ovately-elliptical	elliptical	elliptical
	length (mm)	5.2–7.2	4.0–6.8	3.9–6.3	4.0–4.8	4.3–6.4
	width (mm)	3.4–4.5	2.0–4.2	2.2–3.4	1.2–1.9	1.8–2.9
Outer tepal	Shape	ovately-elliptical	ovately-elliptical	ovately-elliptical	ovate-oblong	ovately-elliptical
	length (mm)	4.8–6.1	3.1–5.0	2.9–5.2	3.7–4.6	3.1–5.2
	width (mm)	2.1–3.7	1.3–3.0	1.1–2.3	1.1–1.7	1.1–2.5
Filament	exsertion	exserted	exserted	exserted	non-exserted	exserted
	length (mm)	6.2–8.4	5.3–8.8	5.0–7.0	3.2–4.4	4.6–6.9
Inner filament	margin	entire	entire	entire	entire	entire or 2-toothed
	shape	narrowly triangular	subulate	subulate	broadened for ca. 1/2 in length	broadened for ca. 1/2 in length
Anther	length (mm)	2.2–2.5	1.7–2.2	1.7–2.0	1.3–1.4	1.5–2.0
	width (mm)	0.9–1.1	0.7–1.0	0.6–0.8	0.6–0.8	0.7–0.9
Ovary	length (mm)	3.2–3.8	2.0–3.4	1.8–2.8	2.1–2.4	2.4–3.1
	width (mm)	3.2–3.7	1.8–3.1	1.5–2.7	1.8–2.0	2.6–2.8
Capsule	length (mm)	5.4–5.6	5.0–5.3	4.8–5.1	3.5–3.7	4.5–5.5
	width (mm)	5.6–5.8	4.5–5.0	4.5–5.0	3.6–4.0	4.5–5.6
Seed	length (mm)	3.7–3.8	3.0–3.3	2.8–3.2	2.0–2.2	3.0–3.5
	width (mm)	2.4–2.6	2.0–2.2	2.0–2.3	1.3–1.5	2.2–2.4
Flowering season		late Sep. to Oct.	Aug. to Sep.	Jul. to Aug.	May to Jul.	Jul. to Aug.
Chromosome number (2n)		2n = 32	2n = 16, 32	2n = 16, 32	2n = 16	2n = 32

### Somatic chromosome numbers

**Figure 3.**
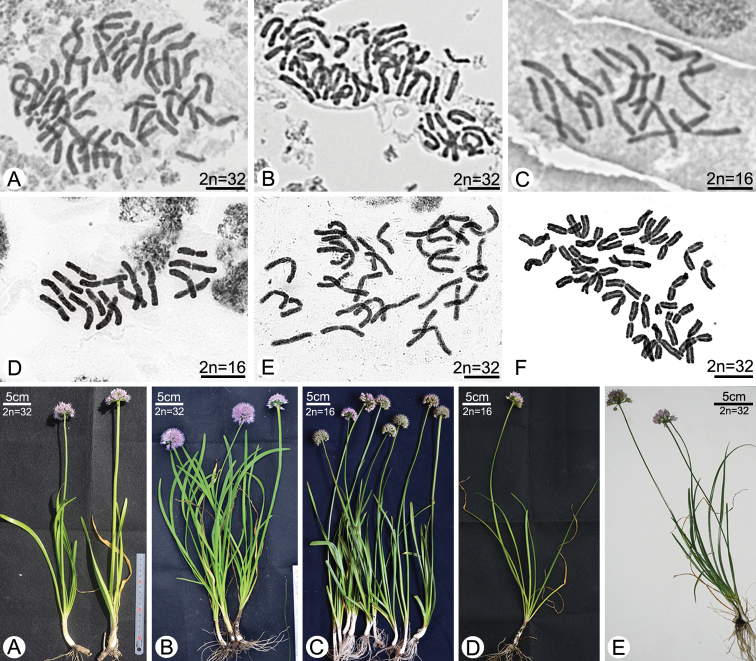
Mitotic metaphase chromosomes and their voucher plants of *Allium* species **A***A. dumebuchum* (*H.J.Choi 190917-01*) **B***A. spirale* (*H.J.Choi 191010-01*) **C***A. spirale* (*H.J.Choi 190910*) **D***A. spurium* (*H.J.Choi 080390*) **E***A. spurium* (*H.J.Choi s.n.*) **F***A. senescens* (*H.J.Choi 080119*, voucher plant: Fig. 2 of Choi and Oh 2010).

The somatic chromosome numbers of *Allium* species investigated were counted as diploid (2*n* = 2*x* = 16; Fig. 3C, D) or tetraploid (2*n* = 4*x* = 32; Fig. 3A, B, E, F). Among studied species, *A. spirale* and *A. spurium* showed polyploidy (Table 3).

### Phylogenetic relationships

Total combined dataset of four chloroplast regions was comprised of 93 samples, including 58 from chloroplast genome. The aligned dataset was 6,046 bp long (4,086 bp in newly sequenced samples) with 556 parsimony-informative site and 4,881 constant site. The dataset consists of *ndhJ*-*trnF*, *trnH*-*psbA*, *psbD*-*trnT*, and *psbJ*-*petA* with 923 bp, 609 bp, 1,121 bp, and 1,095 bp, respectively.

**Figure 4.**
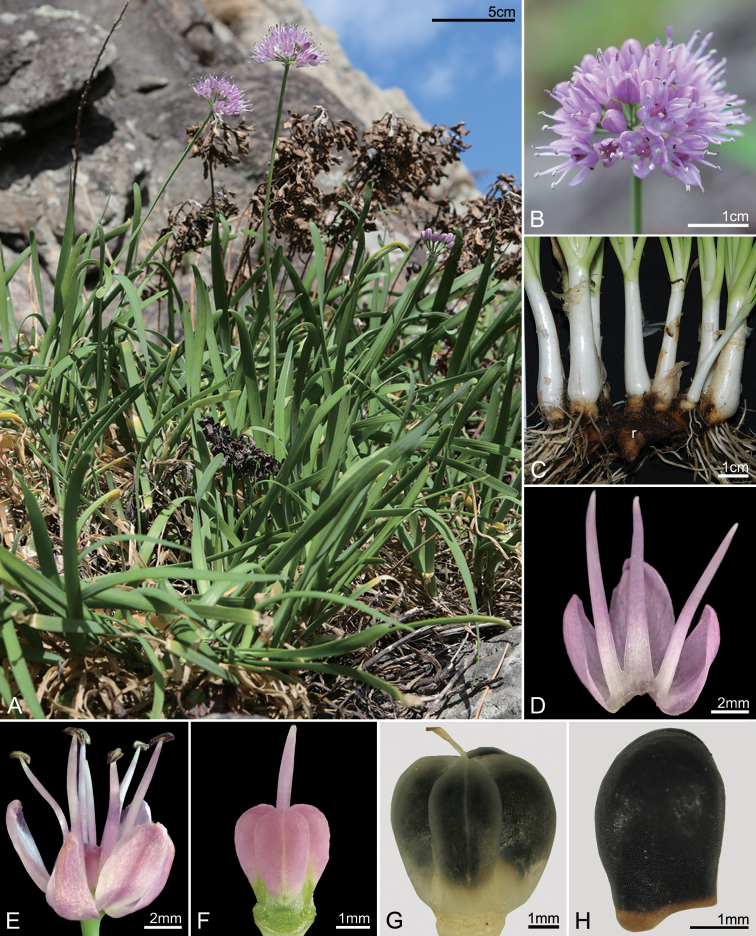
*Allium dumebuchum***A** habit **B** inflorescence **C** underground structure (r = rhizome) **D** tepal and filament arrangement **E** Flower **F** pistil **G** capsule **H** seed. Photos by H.J.Choi: *H.J.Choi 201008-001* (**A, B, D–F**) and *H.J.Choi 070001* (**C, G, H**).

Our phylogenetic tree revealed a similar topology, not showing distinct monophyly, to the previous study (Li et al. 2010; Hauenschild et al. 2017). Nevertheless, subgen. Rhizirideum is monophyletic, despite subgen. Cepa and *Allium* being polyphyletic (Fig. 6). Section Rhizirideum especially constructed a clade supported high bootstrap value (Fig. 6). Allium species in section Rhizirideum, excluding *A. dumebuchum*, dispersed to several clades, showing a confusing phylogenetic relationship. Especially, *A. dumebuchum* revealed monophyly in the tree with high support value and specific morphological characters (Figs 1, 2 and 4), even though it does not show a distinct phylogenetic relationship among the species in section Rhizirideum.

### Taxonomic treatment

#### Key to the species of Allium section Rhizirideum in South Korea and northeastern China

**Table:** 

1a	Leaf sheaths buried under ground; leaf blades leathery, lustrous; perianths campanulate; inner tepals ovate-elliptical; inner filaments entire at margin	**2**
1b	Leaf sheaths exposed above ground; leaf blades fleshy, glaucous; perianths radially spreading; inner tepals elliptical; inner filaments entire or toothed at margin	**3**
2a	Leaf blades 4–10 mm wide; scapes clearly flattened-winged in cross-section	***A. spirale***
2b	Leaf blades 1.5–4 mm wide; scapes rhomboid in cross-section	***A. spurium***
3a	Leaf blades 2.8–4.5 mm wide; scapes subterete in cross-section, 11.7–20.5 mm long; inner tepals 4.0–4.8 mm long, 1.2–1.9 mm wide; outer tepals 3.7–4.6 mm long, 1.1–1.7 mm wide; filaments non-exserted, 3.2–4.4 mm long; capsules 3.5–3.7 mm long, 3.6–4 mm wide; seeds 2.0–2.2 mm long, 1.3–1.5 mm wide; flowering from May to July (2*n* = 2*x* = 16)	***A. minus***
3b	Leaf blades 3.8–15 mm wide; scapes subterete to rhomboid in cross-section, 23.4–70 mm long; inner tepals 4.3–7.2 mm long, 1.8–4.5 mm wide; outer tepals 3.1–6.1 mm long, 1.1–3.7 mm wide; filaments exserted, 4.6–8.4 mm long; capsules 4.5–5.6 mm long, 4.5–5.8 mm wide; seeds 3.0–3.8 mm long, 2.2–2.6 mm wide; flowering from July to October (2*n* = 4*x* = 32)	**4**
4a	Scapes rhomboid in cross-section; perianths light purple; inner filaments narrowly triangular, entire at margin; inner tepals 3.4–4.5 mm wide; ovaries 3.2–3.7 mm wide; flowering from late September to October	***A. dumebuchum***
4b	Scapes subterete in cross-section; perianths pale purple; inner filaments broadened for ca. 1/2 in length, entire or 2-toothed at margin; inner tepals 1.8–2.9 mm wide; ovaries 2.6–2.8 mm wide; flowering from July to August	***A. senescens***

##### Allium dumebuchum

Taxon classificationPlantaeAsparagalesAmaryllidaceae

H.J.Choi sp. nov.

33B1FC43-6CF2-5B81-AFB3-A0D18D3ABDC7

urn:lsid:ipni.org:names:77216563-1

Figs 1A–E , 4

###### Diagnosis.

This new species is morphologically similar to *A. senescens* due to its habits. However, it is clearly distinguished from *A. senescens*, particularly by its rhomboid scapes in cross-secion (vs. subterete), light purple perianth color (vs. pale purple), entire and narrowly triangular inner filaments (vs. sometimes toothed and broadened for ca. 1/2 in length), and flowering season from late September (vs. from July).

###### Type.

South Korea. Gyeongbuk: Ulleung-gun, Namyang, 37.46702N 130.83665E, elev. 11m, 8 Oct 2020 [fl], H.J.Choi 201008-001* (Holotype: KH; Isotypes: CWNU, KB, KIOM).

###### Description.

Herbs hermaphroditic. Rhizomes clearly elongated, thick and branched, oblique to horizontal, 14.8–55.4 mm long. Bulbs clustered, cylindrically conical, 9.6–15 mm in diam.; tunics membranous, smooth, white. Leaves 4–9; sheaths slightly exposed above ground, 4–7.8 cm long; blades ascending, slightly tortuous, linear, flat and solid in cross-section, flesh, 19.5–38 cm × 3.8–13 mm, apex obtuse to rounded. Scapes rhomboid and solid in cross-section, drooping before flowering, 23.4–49 cm × 2.5–5.6 mm. Inflorescences umbellate, subglobose, 23–41.5 × 37–53 mm, 48–113 flowered; pedicels terete, subequal in length, 9.8–11.2mm long; bracts 3.2–5 mm long. Flowers bisexual; perianth semi-radially spreading, light purple; inner tepals longer than outer ones, elliptical, apex obtuse, 5.2–7.2 × 3.4–4.5 mm; outer tepals ovately elliptical, apex obtuse, 4.8–6.1 × 2.1–3.7 mm; filaments exserted, 6.2–8.4 mm long, margin entire; inner filaments narrowly triangular; anthers elliptical, reddish, 2.2–2.5 × 0.9–1.1 mm long; ovary obovoid, reddish, 3.2–3.8 × 3.2–3.7 mm, ovules 2 per locule; style terete, exserted; stigma smooth. Capsules cordiform, trigonous, 5.4–5.6 × 5.6–5.8 mm. Seeds oval, semi-circular in cross-section, 3.7–3.8 × 2.4–2.6 mm.

###### Phenology.

Flowering from late September to October; fruiting from late October to November.

###### Distribution and habitat.

**Figure 5.**
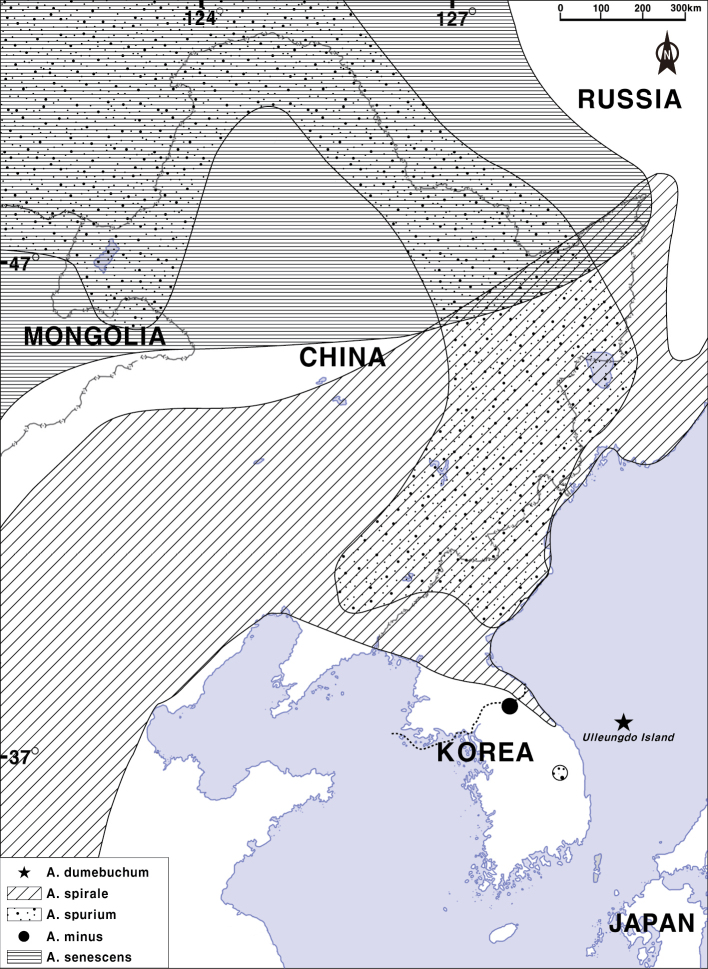
Distribution map of *Allium dumebuchum* and its related species section Rhizirideum in Korea and northeastern China (revised from Sinitsyna et al. 2016).

Endemic to South Korea (Ulleung-do Island; Fig. 5). Open slope of rocky area.

###### Etymology.

The specific epithet, “*dumebuchum*” is based on the name of traditional vegetable for this species in South Korea.

###### Vernacular name.

The Korean name of the new species is “Du-me-bu-chu (두메부추)”.

###### Conservation status.

The new species is endemic to Ulleungdo Island, and usually grows along the coast at altitudes of -23–171m a.s.l. From the present study, the extent of occurrence (EOO) and the area of occupancy (AOO) of this species have been calculated to be 47,683 km^2^ and 48 km^2^, respectively. Currently, there is no information on population size and trend data. However, this new species is only known from a single location of Ulleungdo Island, and mainly occurs on the coast which is critically threatened by extensive construction and repair of coastal roads (Choi et al. 2012a). Therefore, decline in habitat area, habitat extent, and quality of habitat for this species have been continuously observed. Thus, *Allium dumebuchum* should be considered as Critically Endangered [CR B1ab(iii)] according to the IUCN Red List categories and criteria (IUCN 2021).

###### Notes.

**Figure 6.**
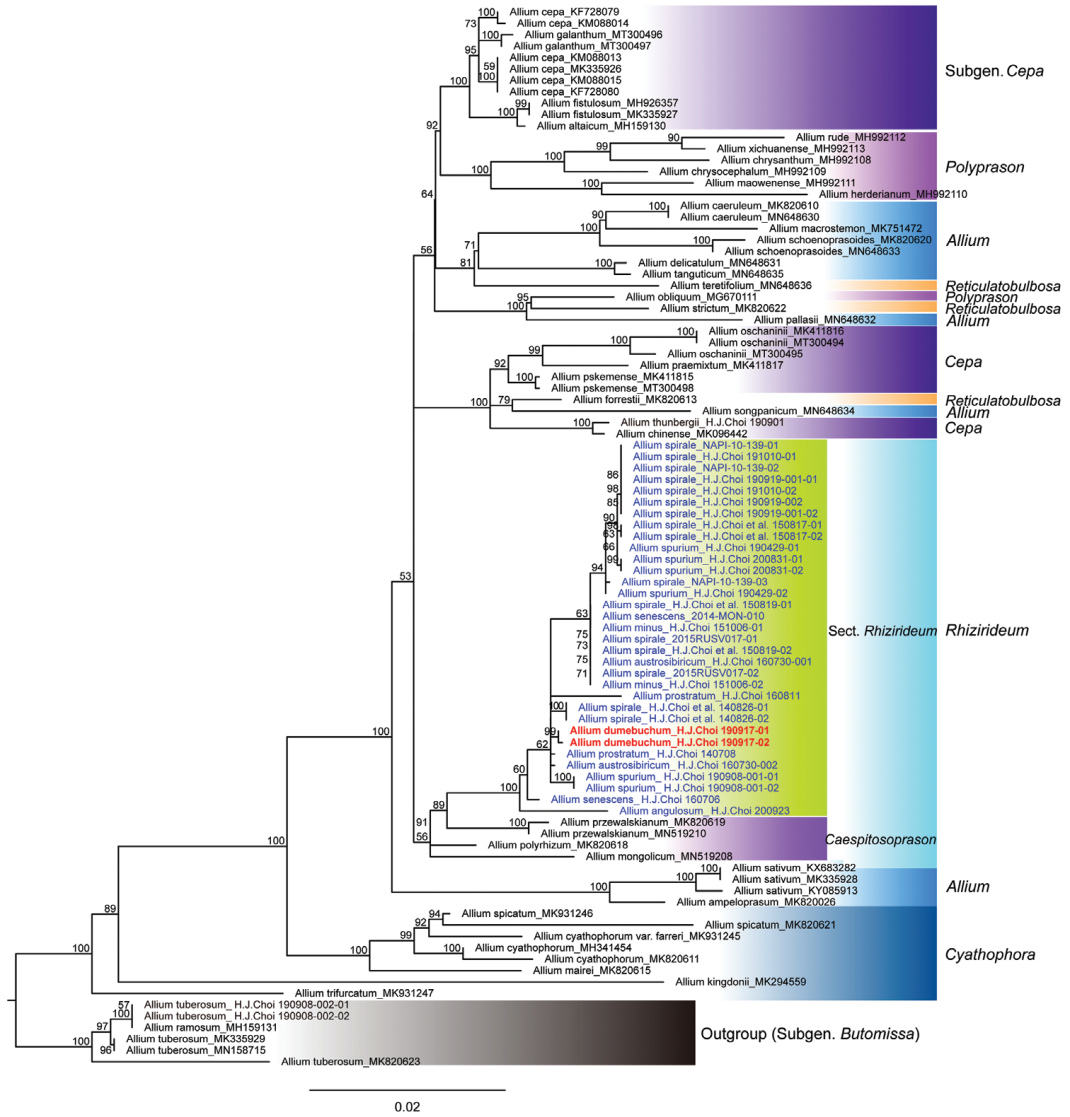
Phylogenetic tree of Allium section Rhizirideum and related taxa based on concatenated alignments of four cpDNA regions (*ndhJ*-*trnF*, *trnH*-*psbA*, *psbD*-*trnT*, and *psbJ*-*petA*). The numbers above branches are bootstrap values (BS > 50%) by maximum likelihood method. The samples of section Rhizirideum and the new species are in blue and red blods, respectively. The accession numbers from Genbank were indicated after the scientific names.

*Allium dumebuchum*, occurring in Ulleungdo Island of South Korea, has usually been misidentified as *A. senescens* (Choi and Oh 2010; Choi and Oh 2011). However, this new species remarkably distinguished itself from its related species of section Rhizirideum (e.g., *A. spirale*, *A. spurium*, *A. minus*, and *A. senescens*) in having clearly bigger floral parts that bloom from late September (Table 3; Fig. 1). The PCA results based on quantitative floral characters of five related species in section Rhizirideum clearly identified *A. dumebuchum* from others (Fig. 2). This new species is a tetraploid (2*n* = 4*x* = 32) taxon along with *A. senescens*, and *A. minus* is a diploid (2*n* = 2*x* = 16), whereas *A. spirale* and *A. spurium* showed polyploidy (Table 3; Fig. 3). Moreover, molecular phylogenetic analyses using chloroplast markers (*ndhJ*-*trnF*, *trnH*-*psbA*, *psbD*-*trnT*, and *psbJ*-*petA*) also clearly indicate that *A. dumebuchum* is genetically distinct from other species of section Rhizirideum (Fig. 6).

###### Additional specimens examined

**(*Paratypes*).** South Korea. Gyeonggbuk: Ulleungdo Isl., Namyang valley, 11 Sep. 2006, *ParkSH 61820* (KH); Ulleungdo Isl., Tonggumi, 26 Sep. 1995, *S-4255* (KH); Ulleungdo Isl., Namyang, 15 Aug. 2009, *Ulleung68-090815-002* (KH); Ulleungdo Isl., Namyang, 22 Aug. 2011, *JMC12750* (KH); Ulleungdo Isl., Namyang, 29 Oct. 2013, *2013KBV091* (KH); Ulleungdo Isl., Namyang, 5 Sep. 2003, *SCHONG2003100* (KH); Ulleungdo Isl., Chusan, 2 Sep. 2009, *JMC11306* (KH); Ulleungdo Isl., Dodong, 18 Sep. 2007, *H.J.Choi 070001* (KH); Ulleungdo Isl., Sadong, 23 Aug. 2005, *1073* (KB); Ulleungdo Isl., Sadong, 23 Aug. 2005, *KH1283* (KB); Ulleungdo Isl., Nari, 25 Sep. 2001, *J.S.Kim s.n.* (KB); Ulleungdo Isl., Hyeonpo, 4 Oct. 2011, *19-1* (KB); Ulleungdo Isl., Nari, 17 Sep. 2019, *H.J.Choi 190917-01* (CWNU); Ulleungdo Isl., Namyang, 8 Oct. 2020, *H.J.Choi 201008-002* (CWNU); Ulleungdo Isl., Namyang, 8 Oct. 2020, *H.J.Choi 201008-003* (CWNU); Ulleungdo Isl., Sadong, 12 Oct. 2005, *NAPI-20101161* (KB); Ulleungdo Isl., 11 Jul. 2013, *H.J.Choi s.n.* (KB); Ulleungdo Isl., 23 Aug. 2005, *1406* (KB); Ulleungdo Isl., 3 Sep. 2008, *SK2008-019-096* (KB); Ulleungdo Isl., 15 Oct. 2009, *ksh84* (KB).

##### Allium spirale

Taxon classificationPlantaeAsparagalesAmaryllidaceae

Willd., Enum. Pl. Suppl. 17 (1814)

2B8E3F2F-F2CC-5711-9B45-6882C8DBFAC5

Fig. 1F–J

###### Type.

Russia (Far East), specimen without collection date and number (Holotype: B photo!).

###### Notes.

*Allium spirale* is occasionally confused with *A. senescens* because of its more or less similar growth habit (Choi and Oh 2011), but the most distinctive characters include clearly flattened-winged scapes (Fig. 1H), campanulate perianth (Fig. 1I) and ovate tepals (Fig. 1J).

###### Specimens examined.

China. Jilin: Gyoha, Ipbeopsan, 2 Sep. 2006, *Jilin23-060902-007* (KH); hunchun, 17 Aug. ?, *S.J.Lee et al. s.n.* (KH); baisan, changbaisan, 22 Aug. 2010, *An-C1273* (KH); Yongjeong, Nampyeong, 8 Sep. 2007, *H.J.Choi & J.W.Han 070014* (KH); Dandong, Aprokgang, 6 Sep. 2007, *H.J.Choi & J.W.Han 070012* (KH); Tungwi, 26 Aug. 1960, *Jilin Teaching Uni. 399* (PE); Tungwi, 14 Jul. 1960, *Yeop 183* (PE); Near O-mu Hsien, 28 Aug. 1931, *H.W.Kung 2195* (PE); Shu-yi Valley, Ching-po Lake, Ning-gu-ta, 5 Sep. 1931, *F.H.Chen 541* (PE); Erdaobaihe, 10 Sep. 2019, H.J.Choi 190910* (CWNU); Wharyoung, 8 Sep. 1959, *700828* (PE). Heilongjiang: Mudanjiangshi, Jingbo lake, 21 Aug. 2001, *ChoiHJ-065* (KH); Harbin, 22 Aug. 2001, *G.W.Park s.n.* (KH); Qinggang, Aug. 1953, *North-eastern group 571* (PE); Saertu, ?, *s.n*. (PE). Liaonong: Xiaodonggou, Benxi, 26 Aug. 1965, *Liu et al. 1319* (PE); Daeryeon, 14 Sep. 1951, *Wang et al. 965* (PE); Héngsan, Daeryeon, 11 Aug. 2008, *B.U.Oh et al*. *s.n.* (CBU). Russia. Primorsky: Mts. Sikhote-Alin, 26 Aug. 2014, *2014CNU001* (KH); Khasan, Lotos lake, 17 Aug. 2015, *2015RUSV017-01* (KH); Bukhta Ekspeditsii, 17 Aug. 2015, *H.J.Choi et al. 150817-01* (KB); Bukhta Ekspeditsii, 19 Aug. 2015, H.J.Choi et al. 150819-01* (KB); Bukhta Ekspeditsii, 26 Aug. 2014, *H.J.Choi et al. 140826-01* (KB); Khasansky, Perevoznaya, 10 Sep. 2013, *5-14* (KB); Khasansky, Shakhterskiy, 11 Sep. 2013, *8-13* (KB); ?, 4 Aug. 2014, *RUS14-3-4* (KB). South korea. Gangwon: Goseong, Ganseong, 12 Oct. 2010, *NAPI-10-139-01* (KH); Goseong, Ganseong, 10 Oct. 2019, *H.J.Choi 191010-01* (CWNU); Gangneung, Gangmun, 19 Sep. 2019, H.J.Choi 190919-001-01* (CWNU); Gangneung, Gangmun, 19 Sep. 2019, *H.J.Choi 190919-002* (CWNU); Gangneung, Yeongok, 02 Oct. 2011, *KYC1965* (KH); Yangyang, Sonnyang, 04 Sep. 2011, *NAPI 2012-0020* (KH); Goseong, Hyeonnae, 15 Sep. 1965, *T.B.Lee et al. s.n.* (KH); Goseong, Ganseong, 10 Sep. 2008, *NAPID2008013* (KB); Goseong, Geojin, 10 Sep. 2008, *J.O.Hyun s.n.* (KB); Gangneung, Sacheon, 15 Nov. 2013, *2013-282* (KB); Goseong, Jugwang, 22 Sep. 2014, *KYC2014-207* (KB); Gangneung, Gangmun, 8 Aug. 2015, *H.J.Choi s.n.* (KB).

##### Allium spurium

Taxon classificationPlantaeAsparagalesAmaryllidaceae

G.Don, Mem. Wern. Nat. Hist. Soc. vi. 59 (1827)

1A1C8F46-D02E-5200-913F-7D0C5E4CA977

Fig. 1K–O

Allium dauricum N.Friesen, Fl. Sibir. (Arac.-Orchidac.) 58 (1987). Type: Russia. Transbaicalia Orientalis, pagum Kyra, in valle fuvii Bukukum, in prato substepposo, 31 Aug. 1964, *G.Peschkova* & *L.Ovczinnicova s.n.* (Holotype: LE!; Isotypes: NSK).

###### Type.

Russia (Siberia, location in doubt). Type specimen not designated (protologue).

###### Notes.

*Allium spurium* is occasionally confused with *A. spirale* because of its more or less similar growth habit, but the most distinctive characters include narrower leaf blades and scapes and smaller floral parts (Table 3; Fig. 1L–N). This species is newly recorded for South Korea, and the new vernacular name ‘Gak-si-du-me-bu-chu’ is given. Besides, Cheongnyangsan of South Korea is the disjunct southernmost limit for geographical distribution of *A. spurium* (Fig. 5).

###### Specimens examined.

China. Jilin: Helong, 8 Sep. 2007, *H.J.Choi s.n.* (KH); Helong, 9 Sep. 2007, *H.J.Choi s.n.* (KH); Baishan, Linjiang, 29 Apr. 2019, *H.J.Choi 190429-01* (CWNU); Erdaobaihe, 08 Sep. 2019, H.J.Choi 190908-001-01* (CWNU). North Korea. Hambuk: Yonsa, 25 Aug. 1958, *C.K.Gen s.n.* (LE). Hamnam: Sinpo, 3 Oct. 2002, *B.U.Oh 020062* (CBU); Hungnam, 21 Aug. 1956, *C.K.Gen s.n.* (LE). Phyonbuk: Huchang, 22 Aug. 1897, *Komarov s.n.* (LE); Jasong, 27 Aug. 1897, *Komarov s.n.* (LE). South Korea. Gyeongbuk: Bonghwa, Cheongnyangsan, 31 Aug. 2020, H.J.Choi 200831-01* (CWNU).

##### Allium minus

Taxon classificationPlantaeAsparagalesAmaryllidaceae

(S.O.Yu, S.Lee & W.Lee) H.J.Choi & B.U.Oh, Brittonia 62(3): 200 (2010)

D83EBFCF-86C6-5FAC-A2C8-892F523088F8

Fig. 1P–T

A. senescens L. var. minus S.OYu, S.Lee & W.Lee, J. Korean Pl. Taxon. 11: 32 (1981) [‘minor’]. **Basionym**.

###### Type.

South Korea. Gangwon: Inje, Wolhaksam-ri, 26 May 1979, *B.S.Gil s.n.* (Neotype: KH!; Oh et al. 2018).

###### Notes.

This species was originally published as a variety of Allium senescens, Allium senescens var. minus ‘minor’. However, this Korean endemic taxon has been revealed as a biologically distinct species. It is remarkably well distinguished from its relatives of the section Rhiziridum by having much narrower and shorter leaf blades and scapes, smaller floral organs, non-exerted filaments and earlier flowering season from May to late July (Table 3; Fig. 1; Choi and Oh 2010; Choi and Oh 2011). Considering these major differences, Choi and Oh (2010) proposed the rank of species for this taxon as more appropriate than that of variety. Although it is cultivated as a vegetable in South Korea, its natural populations are only known from the type locality so far (Fig. 5). However, this species proved to have been extinct in the natural habitat in this study.

###### Specimens examined.

South Korea. Gangwon: Inje, 26 May 1979, *B.S.Gil 0022887* (KWNU); Inje, ?, *W.T.Lee 0022892* (KWNU); Inje, Wolhaksam-ri, 18 May 2008, H.J.Choi 080063* (KH). Gyeonggi: Yangju, Jangheung, 6 Oct. 2015, *H.J.Choi 151006-01* (CWNU).

##### Allium senescens

Taxon classificationPlantaeAsparagalesAmaryllidaceae

L., Sp. Pl. 1: 299 (1753)

ED42E485-5B1C-5045-B2A4-D9140B36F97C

Fig. 1U–Y

Allium pseudosenescens H.J.Choi & B.U.Oh, Brittonia 62(3): 200 (2010). Type: China. Heilongjiang, Tahe, Talin Linchang, *H.J.Choi 080119* (Holotype: KH!; Isotypes: KH!).

###### Type.

Russia. From Siberia (forebaical region), *LINN 419.25* (Lectotype: LINN photo!).

###### Notes.

*Allium senescens*, originally described from the Baikal area of Russia, is certainly one of the most popular ornamental *Allium* species of the world, and is naturally distributed in southern Russia, Mongolia and north-eastern China (Sinitsyna et al. 2016; Sinitsyna and Friesen 2018). The existing records of this species in South Korea (Choi and Oh 2010; Choi and Oh 2011) are all the result of misidentification of herbarium materials, the identity of which we have verified to be *A. dumebuchum*. *Allium pseudosenescens* is newly proposed as an additional synonym of *A. senescens* in this study.

###### Specimens examined.

China. Heilongjiang: ?, 1959, *Wang 163* (PE); Tahe, Talin Linchang, 31 Jul. 2008, *H.J.Choi 080119* (KH); Xifeng Linchang, Tahe, 1 Aug. 2008, *H.J.Choi 080278* (KH); Dashinganryeong, Aug. 1954, *Linxingzu 07577* (PE). Mongolia. Bulgan, Khogno Khaan Mountain Nature Reserve, 29 Jul. 2000, *Sun Byung-Yun 32008* (KH); Sukhbaatar, Tumentsogt, 17 Jul. 2011, *Mongolia_V2012007* (KH); Ulaanbaatar, Sanzai, 09 Jul. 2014, *2014-MON-010* (KB); Tuv, Mungunmorit, 06 Jul. 2016, H.J.Choi 160706* (CWNU); sanzai, 8 Jul. 2014, *2014-MON-010* (KB).

## Supplementary Material

XML Treatment for Allium dumebuchum

XML Treatment for Allium spirale

XML Treatment for Allium spurium

XML Treatment for Allium minus

XML Treatment for Allium senescens
